# Physical structure of the environment contributes to the development of diversity of microalgal assemblages

**DOI:** 10.1038/s41598-024-63867-2

**Published:** 2024-06-12

**Authors:** Áron Lukács, Sándor Szabó, Enikő T-Krasznai, Judit Görgényi, István Tóth, Viktória B-Béres, Verona Lerf, Zsuzsanna Nemes-Kókai, Gábor Borics

**Affiliations:** 1 Institute of Aquatic Ecology, Functional Algology Research Group, HUN-REN Centre for Ecological Research, 18/C Bem Sqr, Debrecen, 4026 Hungary; 2 Department of Ecology, University of Debrecen, 1 Egyetem Sqr, Debrecen, 4032 Hungary; 3 Department of Biology, University of Nyíregyháza, PO Box 166, Nyíregyháza, 4401 Hungary; 4 Juhász-Nagy Pál Doctoral School of Biology and Environmental Sciences, University of Debrecen, 1 Egyetem Sqr, Debrecen, 4032 Hungary

**Keywords:** Ecology, Ecology, Environmental sciences, Limnology

## Abstract

Aquatic macrophytes form a three dimensional complex structure in the littoral zones of lakes, with many physical, chemical and biological gradients and interactions. This special habitat harbours a unique microalgal assemblage called metaphyton, that differs both from the phytoplankton of the pelagial and from the benthic assemblages whose elements are tightly attached to the substrates. Since metaphytic assemblages significantly contribute to the diversity of lakes’ phytoplankton, it is crucial to understand and disentangle those mechanisms that ensure their development. Therefore, we focused on the question of how a single solid physical structure contribute to maintaining metaphytic assemblages. Using a laboratory experiment we studied the floristic and functional differences of microalgal assemblages in microcosms that simulated the conditions that an open water, a complex natural macrophyte stand (*Utricularia vulgaris* L.), or an artificial substrate (cotton wool) provide for them. We inoculated the systems with a species rich (> 326 species) microalgal assemblage collected from a eutrophic oxbow lake, and studied the diversity, trait and functional group composition of the assemblages in a 24 day long experimental period. We found that both natural and artificial substrates ensured higher species richness than the open water environment. Functional richness in the open water environment was lower than in the aquaria containing natural macrophyte stand but higher than in which cotton wool was placed. This means that the artificial physical structure enhanced functional redundancy of the resident functional groups. Elongation measures of microalgal assemblages showed the highest variation in the microcosms that simulated the open water environment. Our results suggest that assembly of metaphytic algal communities is not a random process, instead a deterministic one driven by the niche characteristics of the complex three dimensional structure created by the stands of aquatic macrophytes.

## Introduction

According to their mode of life, microalgae are usually classified as either planktic or benthic. The first term encompasses those algae that spend their whole life in the pelagic water and do not need hard substrate for their survival and reproduction. The other term refers to those taxa that grow on solid surfaces and have a sessile life form. However, this sharp separation was challenged in the middle of the last century. Hutchinson^1^ in his seminal paper “The paradox of the plankton” pointed out that with the exception of the ocean phytoplankton, many of the planktic algae do not form a self-perpetuating community, since during a given period of the year several species may occasionally have a benthic life form^2–4^. Thus, in freshwater ecosystems the movement of species from the littoral zone to the open water may potentially help to maintain high phytoplankton diversity. The process is not identical to that occurring in the littoral, where physical disturbances detach benthic algae from the substrate and carry them to the open water^5^. Furthermore, it has been also demonstrated that in eutrophic lakes, the macrophyte-dominated state does not necessarily result in a clear-water state, because among the submerged macrophytes rich and abundant algal assemblages can develop that are compositionally different from the phytoplankton of the adjacent open water^6^. These differences are attributable to the special environment that macrophytes create in their immediate surroundings. Submerged, free-floating and floating leaved aquatic plants have a variety of effects on the phytoplankton^7^, by altering light and nutrient availability^8–10^, providing shelter to zooplanktic grazers^11–13^ or by producing and releasing allelopathic substances^14–17^. The allelochemicals are mostly specific to particular organisms^18,19^ and can be released to the water inhibiting microalgae, or can be concentrated on surfaces modifying the direct cell–cell contacts^20^. The above mentioned physical, chemical and biological effects create an array of microgradients among the stems and leaves of macrophytes enabling the development of diverse microalgal assemblages called metaphyton^2^.

In the temperate zone, species of lake phytoplankton assemblages are recruited from various sources, such as from low abundance overwintering populations or from resuspension of resting forms settled in the sediment^21,22^. However, in lakes and ponds with extended littoral vegetation the metaphyton represents a potential source of species recruitment into the pelagial assemblages^23^. Field observations^24^ and lake enclosure experiment^25^ revealed that there is a flow of species from the metaphyton towards the pelagial water, enhancing its taxonomic and functional diversity. A proper understanding of this process requires the exploration and evaluation of those mechanisms that are considered relevant for maintaining the high microalgal diversity of the metaphyton. As it was mentioned above submerged macrophytes create special microhabitats that differ from the open water in their physical, chemical and biological characteristics. Both physical and chemical cues of macrophytes are important in site selection of benthic (and metaphytic) organisms^26^. While the chemical interactions between the substrate and resident species are well documented in the literature, much less attention has been paid to the role of the seemingly simpler physical structures^27^. The physical constraints of the aquatic systems (density, viscosity, current velocity) have a direct impact on the physical dimensions of microorganisms like their size and shape^28^. Since the physical conditions prevailing in the macrophytes stands of the lakes’ littoral are different from that of the open water areas, it is reasonable to suppose that these differences appear in the size and shape variation of the resident species. Therefore, in this research, we focused on the questions of how the physical structure of macrophytes influences the diversity and trait composition of metaphytic algal assemblages. To answer this question, we set up a “ceteris paribus” experimental design to disentangle the effect of physical structures from the simultaneous effect of other (chemical and biological) factors. We performed microcosm experiments using living macrophytes as natural-, cotton wool as artificial structures, in various experimental setups, and after inoculating the microcosms with species rich algal assemblages, we studied the survival of microalgal species during a 24 day culture period.

Since submerged physical structures can provide temporary shelter for a variety of microalgae, we hypothesised that:

(i) Physical structure of submerged macrophytes alone is sufficient to maintain high microalgal species richness in aquatic systems.
(ii) Species and functional richness would show differences between the natural and artificial substrates.
(iii) Morphological trait composition of the observed taxa would be different in the various experimental setups.

## Materials and methods

### Experimental design

The metaphyton similarly to the assemblages of the planktic and benthic realms shows systematic changes during the year, and attains its higher diversity in the second half of the vegetation period^25^. Species rich metaphytic assemblages take several weeks to develop which makes its experimental study a challenging task. Therefore, instead of investigating how a rich assemblage can develop on/among natural and artificial physical structures, we studied how these physical structures contribute to maintaining high microalgal diversity, in comparison to each other and to the open water.

Using a microcosm experiment, we studied how the trait and species composition changes over 24 days in aquaria containing (i) artificial structure (cotton wool), (ii) natural structure (*Utricularia vulgaris* L), and (iii) algal inoculum free from substrate. It is known that exceptionally high phytoplankton and periphyton diversity can develop on and among the stands of *Utricularia* spp*.*^29,30^. This carnivorous plant does not produce allelopathic substances^31^ and is widespread in Hungarian and European waters^32,33^, making it suitable for studies.

To make an algal inoculum (hereafter as A), microalgae were collected from a eutrophic oxbow lake (Nagy–Morotva, NE Hungary–48^○^07ʹ12.30ʹʹ 21^○^ 27ʹ59.34ʹʹ; physical and chemical variables of the oxbow lake are shown in Supplementary Table 1). We collected plankton samples in the littoral zone of the oxbow lake and concentrated them by plankton net of 10 µm mesh size. This sample was enriched with additional species obtained by squeezing a handful of *Utricularia vulgaris* and *Salvinia natans* L. plants. We used this concentrated algal suspension as algal inoculum (A) in the experiment. To estimate the number of species of the algal inoculum we used Chao’s sample-based extrapolation curve^34^.

**Figure 1:**
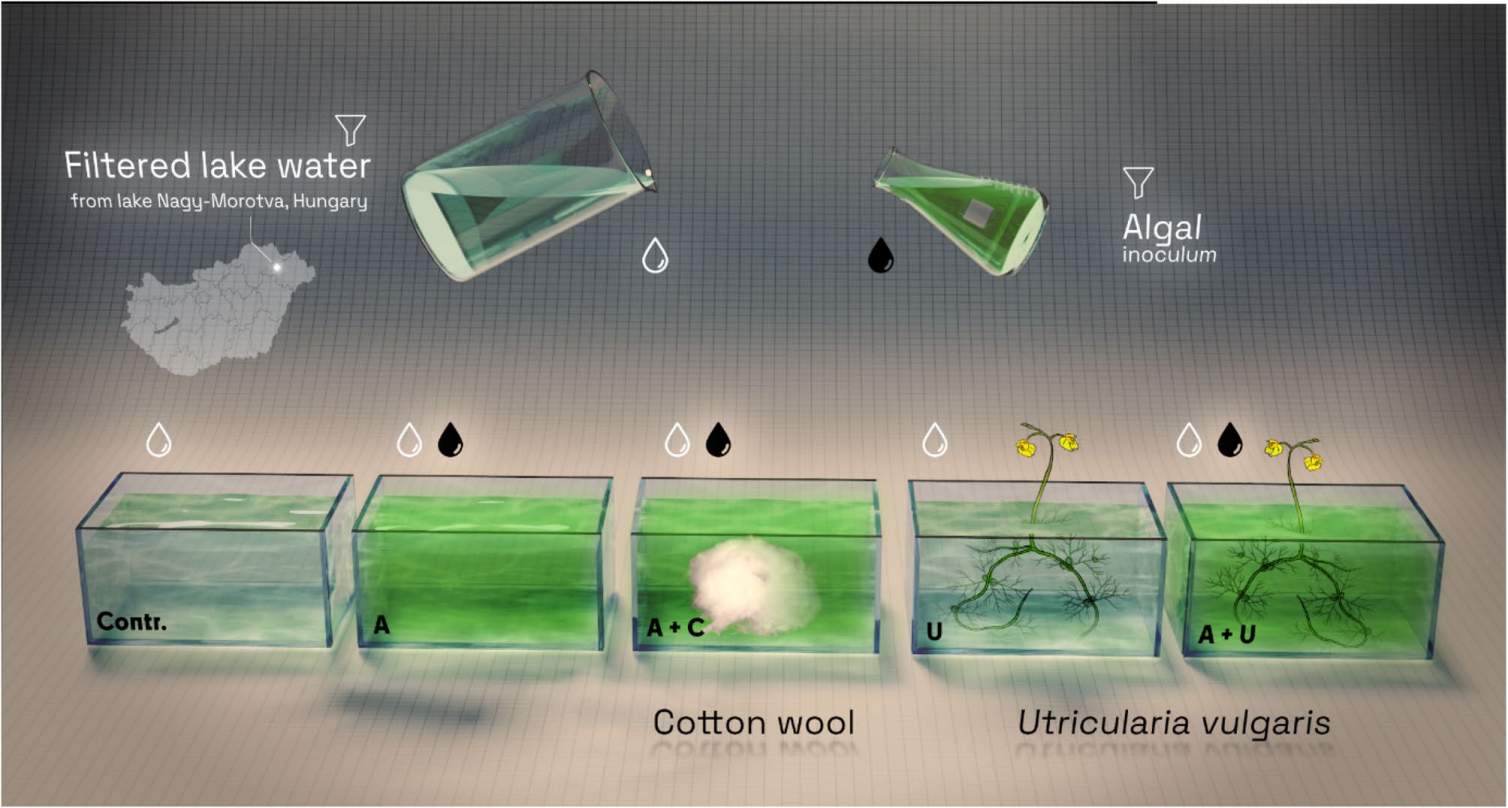
A schematic view of experimental setup.

A total of five treatments were applied, each in three biological replicates, so the experimental design consisted of 15 pieces of plastic aquaria (2 L) (Fig. 1; Table 1): (1) control (Contr.), (2) 20 ml algal inoculum (A), (3) A and 10 g cotton wool (A+C), (4) 10 g fresh mass of *Utricularia vulgaris* (U), (5) A and 10 g fresh mass of *Utricularia vulgaris* (A+U) (Fig. 1). Water from the oxbow lake filtered by 10 µm mesh plankton net was used as a standard growing medium and added to each aquarium. The Contr. setup contained only the standard growing medium and was used as a control for aerial contamination, while the use of rinsed *U. vulgaris* (U) alone was necessary to detect the algae that survived washing. We removed water from the surface of the *Utricularia* plants by using a salad centrifuge and then placed shoots of *Utricularia* (10–25 cm length) in the aquaria U and A+U. Initial biomass was 10 +/− 0.1 g fresh weight that ensured 100% coverage within the aquaria. To mimic the delicate structure of submerged aquatic plants, 10 +/− 0.1 g cotton wool was added to the aquaria (C). The A setup contained the growing medium and algal inoculum, this setup was used to simulate open water conditions. The A+U setup mimicked conditions in the metaphyton. The microcosms were kept in water bath at controlled temperature of (22–24 °C), and incubated in a 12:12 h L/D regime at 80 µmol m^−2^ s^−1^ PAR photon flux density. Illumination was obtained by using 400 W metal halogen lamps.

**Table 1:** Experiment design: components of each treatment with abbreviations*.*

Treatment	Abbreviation
Control	Contr
Algal inoculum	A
Algal inoculum + cotton wool	A+C
*Utricularia vulgaris*	U
Algal inoculum + *Utricularia vulgaris*	A+U

To avoid rapid sedimentation of algae (and their consumption by benthic grazers) in the microcosms, using a 5 ml pipette we gently mixed the water at the bottom of each aquaria daily with pumping it in and out three times each day of the experiment and additionally before each sampling occasion. After inoculation we sampled the communities for 24 days (on days 0, 2, 4, 8, 16, 24) means that microflora of 78 samples were collected for further analyses. During the sampling of the 15 microcosms we collected 5 × 5 ml of water by pipette from different parts of each aquarium (also from parts with high plant/cotton wool density), so that a sample from one aquarium had a volume of 25 ml. The samples were fixed with Lugol’s solution. For microscopic analyses 5 ml of the samples were poured into 5 ml counting chambers and allowed to settle for 24 h. Samples from biological replicates were counted and digitalized separately.

We studied the phytoplankton samples at ×100 and ×400 magnification using a Zeiss AxioObserver inverted microscope and counted at least 400 units (cells, colonies and filaments) in every sample according to the Utermöhl method (1958)^35^. For biovolume estimation, we measured the linear dimensions of 20 specimens of each taxon and calculated their biovolume and surface area using realistic 3D models^36^. Accepted names of phytoplankton species were based on the AlgaeBase^37^.

### Functional properties of metaphytic species

Besides revealing taxonomic differences among the experimental setups, we also studied the functional response of microalgal assemblages to the different environments. We used both functional group-based and functional trait-based approaches. We assigned the observed species into functional groups (hereinafter FGs) according to Reynolds et al., Borics et al. and Padisák et al.^38–40^. We defined Functional richness as the number of FGs in the samples. To assess the functional redundancy of the samples we used the following Eq. (1)

1 $${\text{FR}}_{{\text{i}}} \,{ = }\,{\text{n}}_{{\text{i}}} {\text{/N}}_{{\text{i}}}$$

where:

FR_i_: functional redundancy of the ith functional group.

n_i_: number of species belonging to the ith functional group in the given sample.

N_i_: total number of species belonging to the ith functional group.

### Quantifying the morphological traits of microalgal cells and colonies

Since we investigate the impacts of the physical structures on the microalgal assemblages, we selected morphological and morphology related size traits; such as relative elongation and specific biovolume of the species^41^. Using these measures, we defined relative elongation (RE) as a rate of difference from a sphere and calculated as the greatest axial linear dimension of the cell/equivalent spherical diameter ratio (Eq. (2))

2 $$RE = \frac{GALD}{{ESD}} = \frac{GALD}{{\sqrt[3]{{\frac{6V}{\pi }}}}}$$

where:

RE: relative elongation.

V: volume of the object.

GALD: greatest axial linear dimension of the cell (or colony).

ESD: diameter of sphere having an equal volume with the given species.

Using the Blender open source graphics application, we created the shape realistic models of each species, and applying the NeuroMorph program tool^42^, we measured the biovolume of the objects and their greatest linear dimensions (GALD).

To assess the size and morphological variability of the assemblages, we created a morphospace where community weighted mean values (CWM) of the RE have been plotted against the CWMs of log-biovolume of each species that occurred in the given assemblage. Since each dot in the morphospace represents one assemblage, the dispersion of dots belonging to a given experimental setup indicates its morphological variation. The dots were surrounded by a convex hull whose area describes this variation.

### Statistical analyses

To compare species and functional richness, functional redundancy and trait composition between treatments we used analysis of variance (ANOVA) for normally distributed variables, and Kruskal–Wallis test when normality was not satisfied. When the results showed significant differences between treatments, Tukey and Dunn’s post-hoc tests were used to further explore differences (normality of the data was also checked). In the case of trait-based analyses, we calculated weighted means (CWM) of traits for each samples and the tests were performed using these CWM values.

Preliminary analyses (visual inspection of changes in number of taxa in time; linear models) indicated rapid changes in the assemblages that were significantly affected by time, especially in the first week of the experiment (Supplementary Table 2). Consequently, in order to highlight compositional differences between treatments and eliminate the effect of time on the results, most of the analyses only included samples from the 8th day of the experiment. However, samples from the 2nd day were incorporated in the variance analyses of species richness and number of functional groups.

Analysis of similarity (ANOSIM) and pairwise permutational analysis of variance (PERMANOVA) were used to test for differences in taxonomic and functional group composition between treatments. Non-metric multidimensional scaling (NMDS) was used to visualise these results.

Linear models were built to reveal the relationship between number of species and functional richness. Since both variables met the normal distribution assumption, correlation coefficients were determined by using Pearson correlation. The significance of difference between the models were tested with Fisher Z-test with the use of the correlation coefficients.

All statistical tests were performed in RStudio^43^.

## Results

### Species richness of the inoculating material

**Figure 2:**
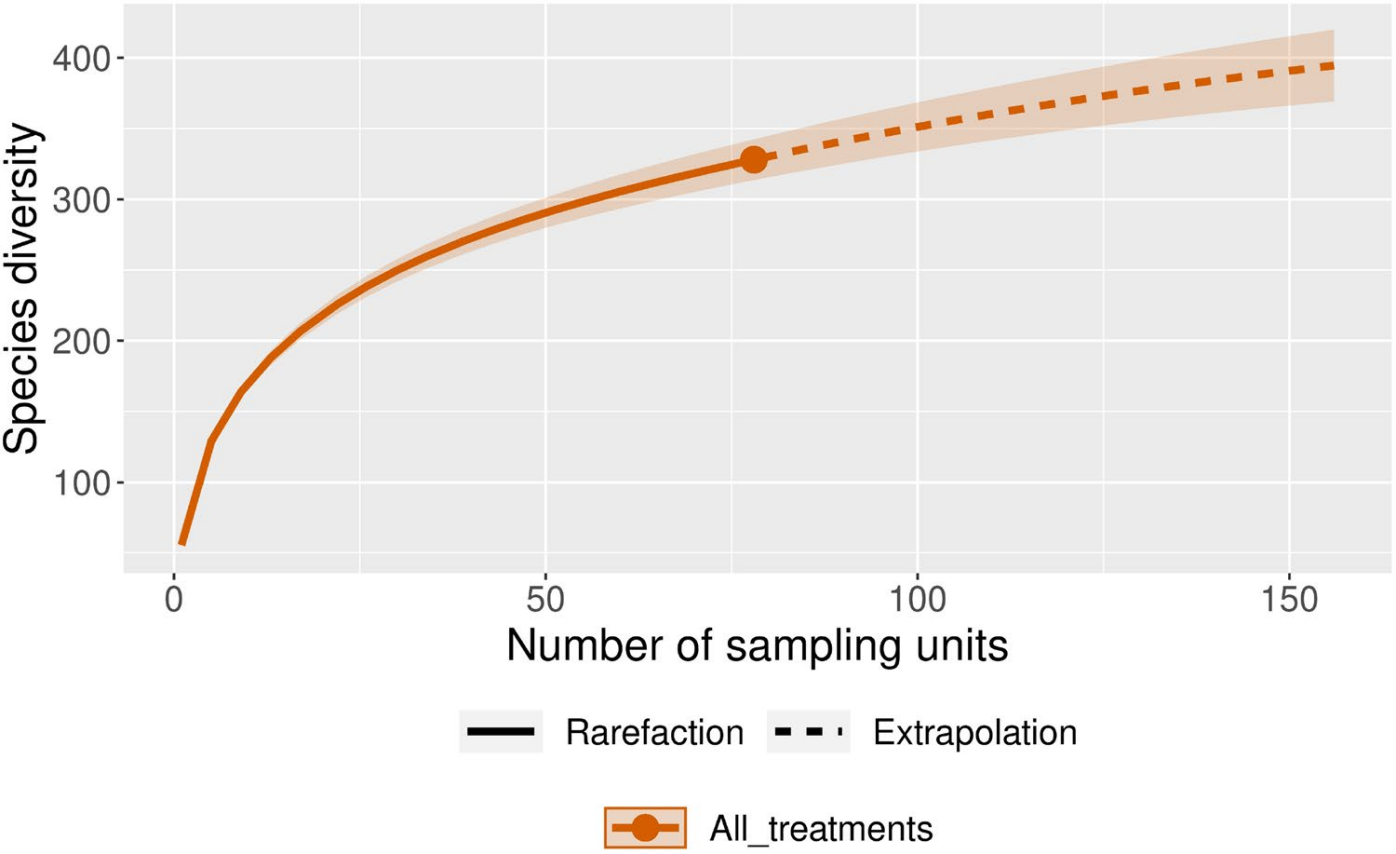
Chao’s species based extrapolation curve to estimate overall species richness of the experimental setups, (N = 78).

We observed 326 species (Supplementary Table 3) in the samples, but the Chao’s sample-based extrapolation curve^34^ was not asymptotic, indicating that the estimated number of species was considerably larger (Fig. 2). Since these kinds of approaches allow a short range extrapolation^44^ applying an extrapolated sample size of twice the number of observed samples the number of species in the experiments should approximate 400.

**Figure 3:**
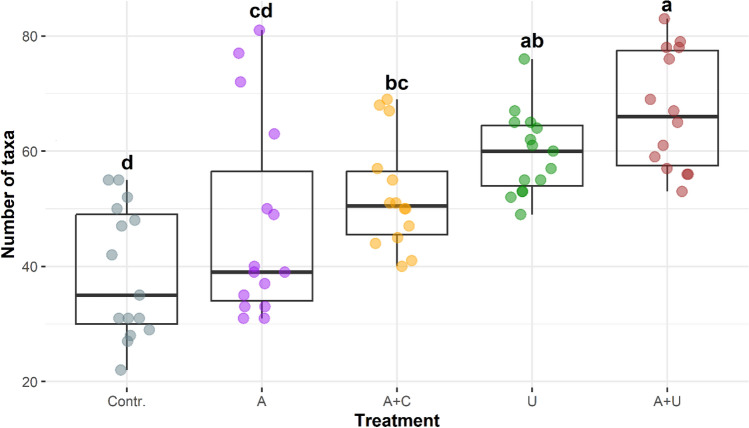
Species richness of the experimental setups from the 2nd day of the experiment. The boxplots visually depict the distribution of data by representing the interquartile range with a box, indicating the median with a bold line inside the box, and displaying the minimum and maximum values through whiskers (points outside the whiskers’ range are outliers). Homogenous groups are indicated with different lowercase letters and based on significant differences of the number of the species among treatments (Tukey’s test, P < 0.05). *Contr.* standard growing medium as a control, *A* standard growing medium with algal inoculum, *A*+*C* standard growing medium with cotton wool and algal inoculum, *U* standard growing medium with *Utricularia vulgaris*, *A*+*U* standard growing medium with *Utricularia vulgaris* and algal inoculum, (N = 75).

Microalgal diversity showed considerable differences in the five experimental setups (Fig. 3). The lowest mean values in species richness appeared in the microcosms that simulated open water situation (Contr. and A). These setups were characterised by larger data dispersion (> 35 species). We observed the most species rich assemblages in those microcosms in which physical structures (*Utricularia* or cotton wool) were present (A+C, U, A+U). This was true for the minimum and mean values. High diversity occasionally developed in the Contr. and A setups.

**Figure 4:**
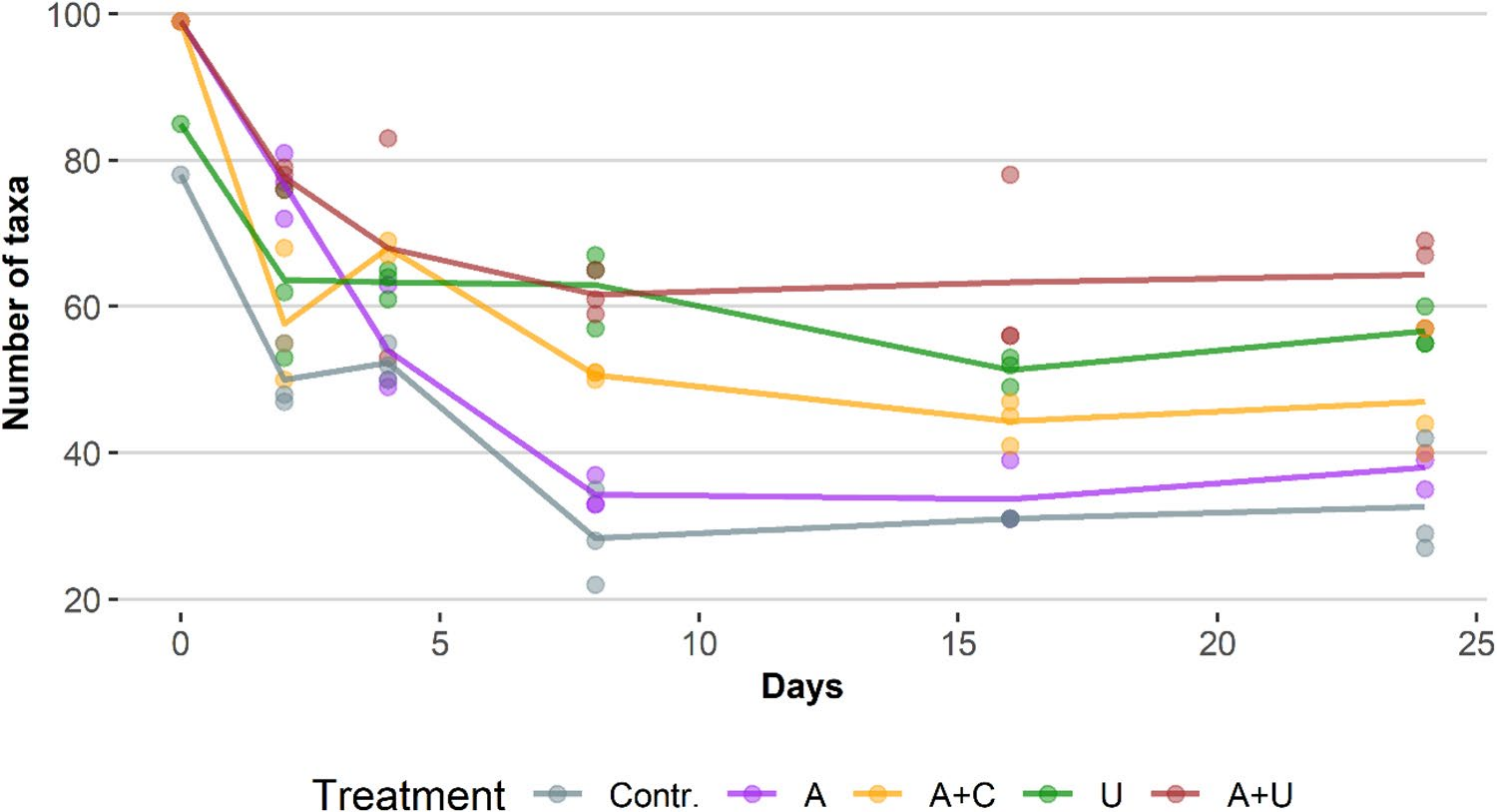
Temporal changes in number of species in the experimental setups through the whole study period. Abbreviations for experimental setups are specified under Fig. 3, (N = 78).

Temporal changes of species richness values showed similar trends in each experimental setup. The species numbers decreased rapidly from the onset of the experiment up to the 8th days, and then stabilized showing little changes until the end of the experiment (Fig. 4). Richness values of microcosms in which *Utricularia* were placed were above, while those of the open water microcosms were below the richness values of cotton wool containing setups.

**Figure 5:**
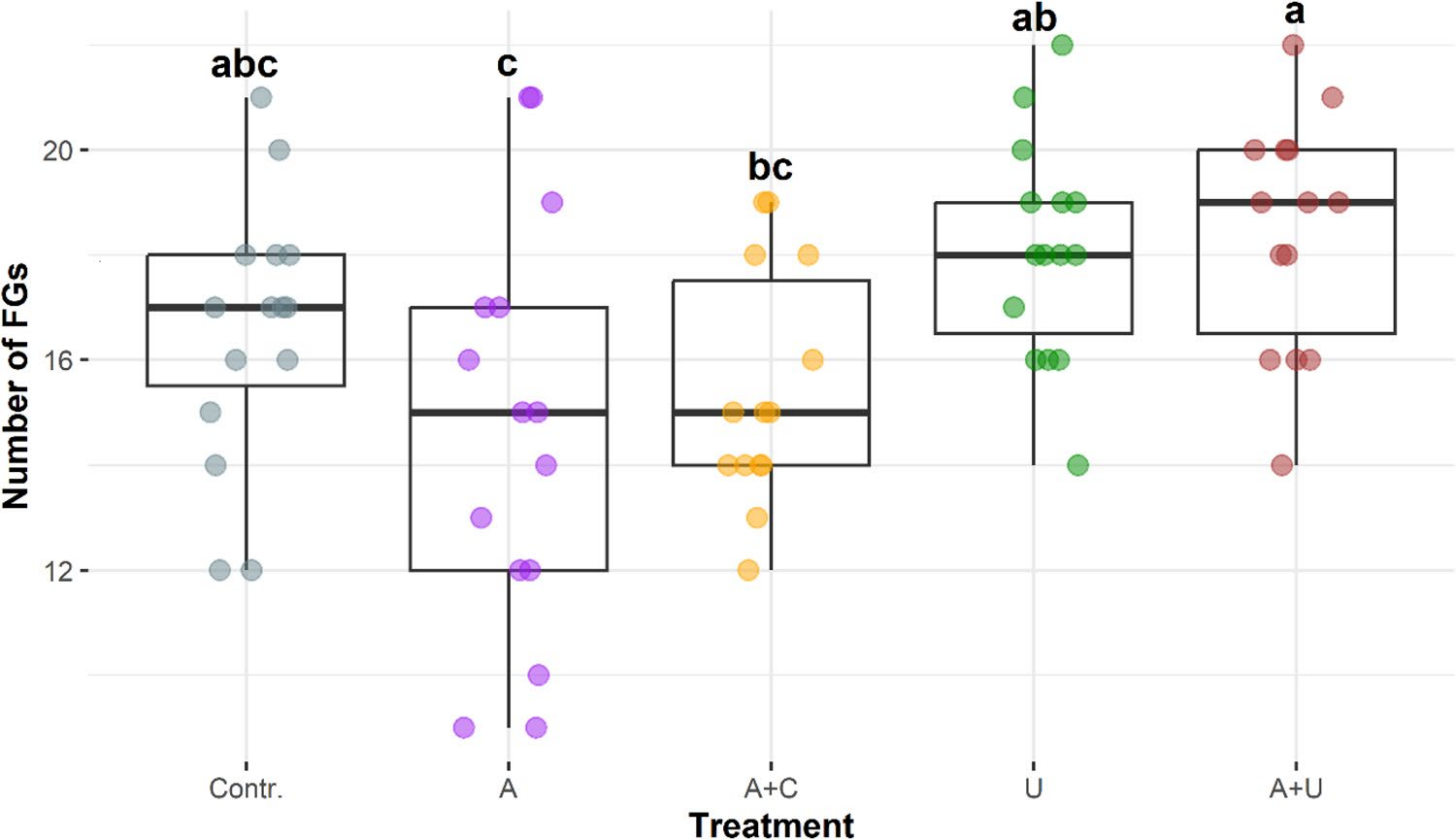
Functional richness as the number of functional groups (FGs) in the experimental setups from the 2nd day of the experiment. The boxplots visually depict the distribution of data by representing the interquartile range with a box, indicating the median with a bold line inside the box, and displaying the minimum and maximum values through whiskers (points outside the whiskers’ range are outliers). Homogenous groups are indicated with different lowercase letters and based on significant differences of the number of FGs among treatments (Tukey’s test, P < 0.05). Abbreviations for experimental setups are specified under Fig. 3, (N = 75).

**Figure 6:**
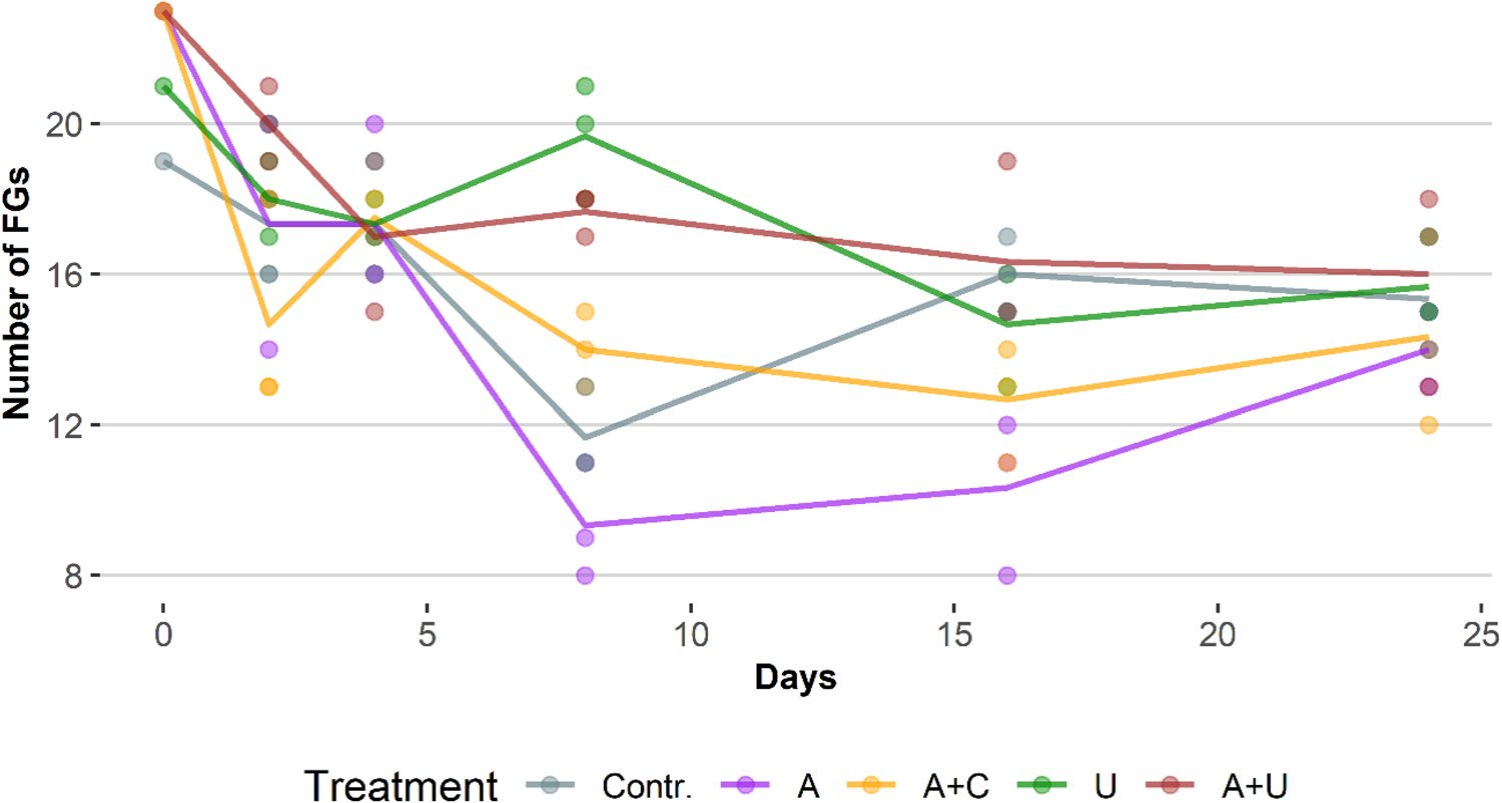
Temporal changes of functional richness (as number of functional groups (FGs)) values through the whole study period. Abbreviations for experimental setups are specified under Fig. 3, (N = 78).

Functional richness of the five experimental setups showed differences in terms of their means and dispersions (Figs. 5 and 6). The microcosm that simulated the open water situation (A) and contained the artificial physical structures (A+C) had the lowest values. Significantly higher functional richness values characterised those microcosms that contained *Utricularia* plants, both the inoculated (A+U) and non-inoculated ones (U). The mean value of microcosms that contained exclusively filtered lake water (Contr.) are positioned in the middle range of functional richness.

Similarly to species richness, functional richness values also fell at the beginning of the experiment, but from the 4th and 8th days different tendencies could be observed among the experimental setups (Fig. 6). Slightly decreasing (A+C, A+U) or increasing tendencies (A), and unpredictable changes (Contr., U) could also be observed.

We found a positive correlation between the species richness and functional richness values (Table 2), however the position of the lines of some microcosm setups suggests asynchronous changes of the two variables. This asynchrony appeared prominently in the case of A and A+C, and can be explained by differences in functional redundancy values (Supplementary Fig. 1). Majority of the FGs contained only few (< 4) species. In the case of these FGs functional redundancy changed unpredictably. However, there were some FGs with a considerable redundancy (> 10), and also with remarkable changes in redundancy values. The X1, J, TIB, F N, X2 functional groups showed a decreasing tendency with time in most of the microcosm setups. The most drastic decline occurred in the A setup, where species richness of the TIB and N groups dropped to low values. However, redundancy values of the N functional group indicated an increasing tendency in the A+C, U and A+U systems, i.e. in those where natural (*Utricularia*), or artificial (cotton wool) substrates were present.

**Table 2:** Results of the correlations between number of species and functional richness.

P-value						Correlation coefficient	R-squared
	Contr.	A	A+C	U	A+U		
Contr.		0.75	0.72	0.8	0.62	0.76	0.57
A	− 0.32		0.96	0.57	0.41	0.81	0.65
A + C	− 0.36	− 0.05		0.55	0.40	0.81	0.66
U	0.25	0.56	0.60		0.81	0.71	0.50
A + U	0.50	0.82	0.85	0.24		0.65	0.43
Z-test statistic							

The cells contain correlation coefficients, R-squared values of the models and the results of the Fisher Z-test (P-values in the upper diagonal of the matrix and Z-test statistics in the lower diagonal), (N = 75).

**Figure 7:**
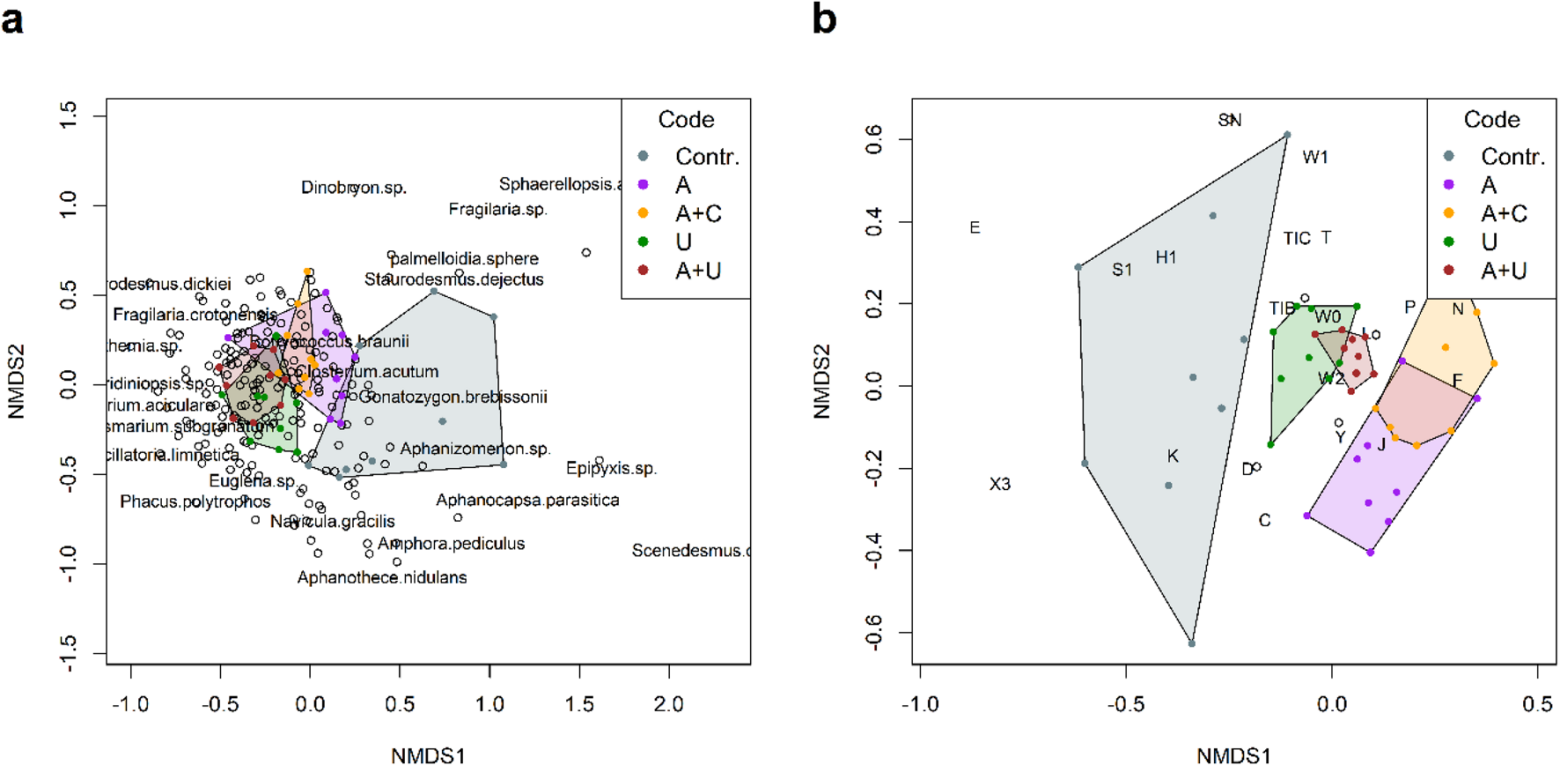
Compositional differences of the experimental setups displayed by non-metric multidimensional scaling in the case of (**a**) species and (**b**) functional groups from the 8th day of the experiment. Abbreviations for experimental setups are specified under Fig. 3, (N = 45).

The species-based NMDS ordination revealed clear separation of communities of the filtrated water (Contr.) from the other microcosm setups (Fig. 7a). Those microcosms that contained *Utricularia* (U, A+U) also separated from the others, but these only marginally differed from each other (Table 3). The assemblages of the microcosms that mimicked the open water (A) and the artificial substrate (A+C) showed some resemblance.

**Table 3:** Results of the species-based pairwise PERMANOVA (upper diagonal) and functional group-based pairwise PERMANOVA (lower diagonal).

Species-based pairwise PERMANOVA					
	Contr.	A	A+C	U	A+U
Contr.		0.01	0.01	0.01	0.01
A	0.01		0.06	0.01	0.01
A+C	0.01	0.01		0.01	0.01
U	0.03	0.01	0.01		0.02
A+U	0.01	0.01	0.01	0.06	
FG-based pairwise PERMANOVA					

Abbreviations for experimental setups are specified under Fig. 3, (N = 45).

The functional group-based NMDS ordination yielded similar results in that the assemblage of the filtrated water (Contr.) differed considerably from that of the other microcosm setups (Fig. 7b). The FG composition of the assemblages in the U and A+U microcosms also showed resemblance. However, in contrast to the species-based composition, the FG-based ordination revealed a significant distinct separation of the inoculated plankton (A) and cotton wool (A+C) assemblages.

### Distribution of morphological traits in the microcosm setups

**Figure 8:**
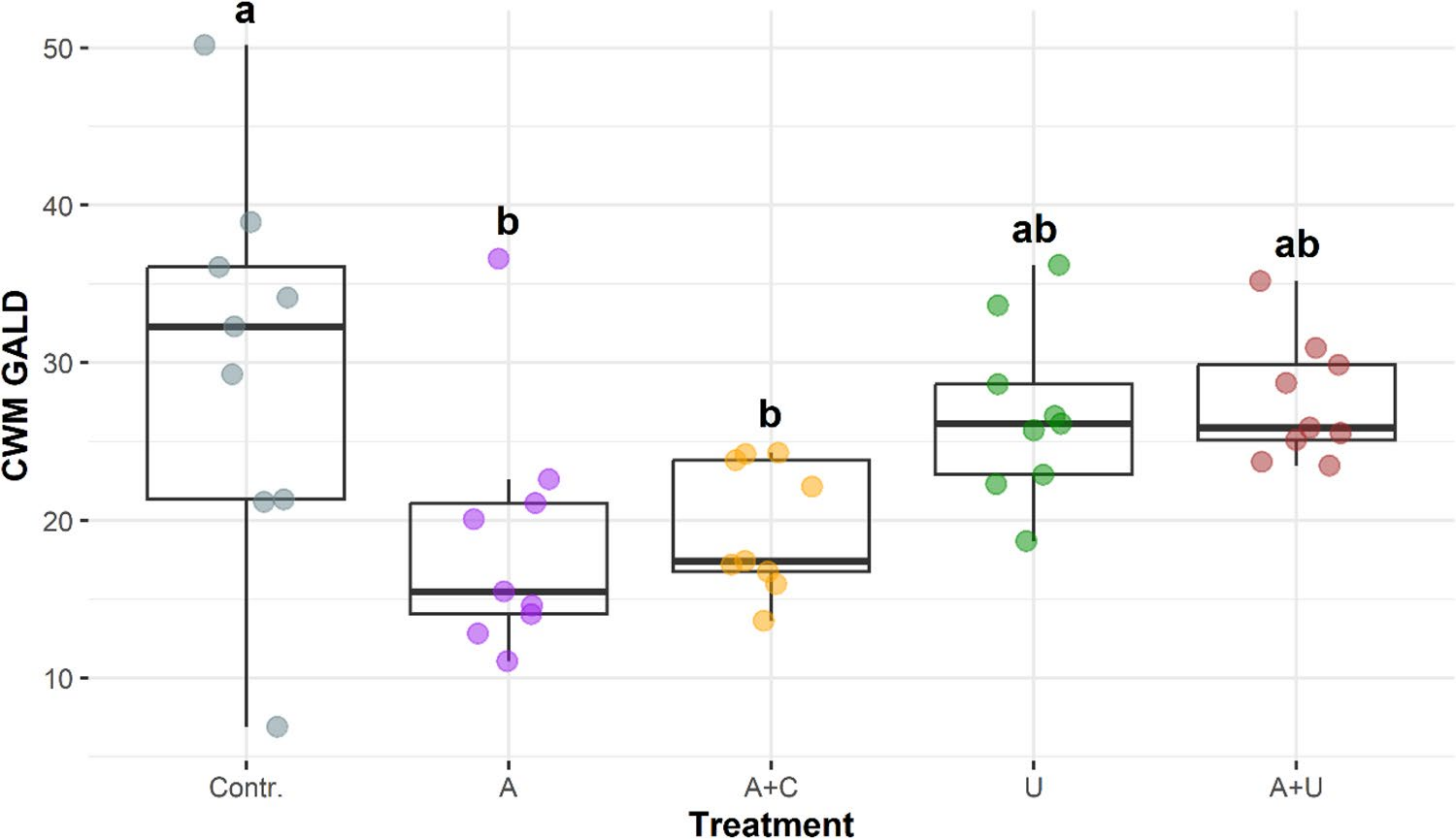
Community weighted means of the greatest linear dimension (CWM GALD) in the experimental setups from the 8th day of the experiment. The boxplots visually depict the distribution of data by representing the interquartile range with a box, indicating the median with a bold line inside the box, and displaying the minimum and maximum values through whiskers (points outside the whiskers’ range are outliers). Homogenous groups are indicated with different lowercase letters and based on significant differences of the CWM values of GALD among treatments (Tukey’s test, P < 0.05). Abbreviations for experimental setups are specified under Fig. 3, (N = 45).

**Figure 9:**
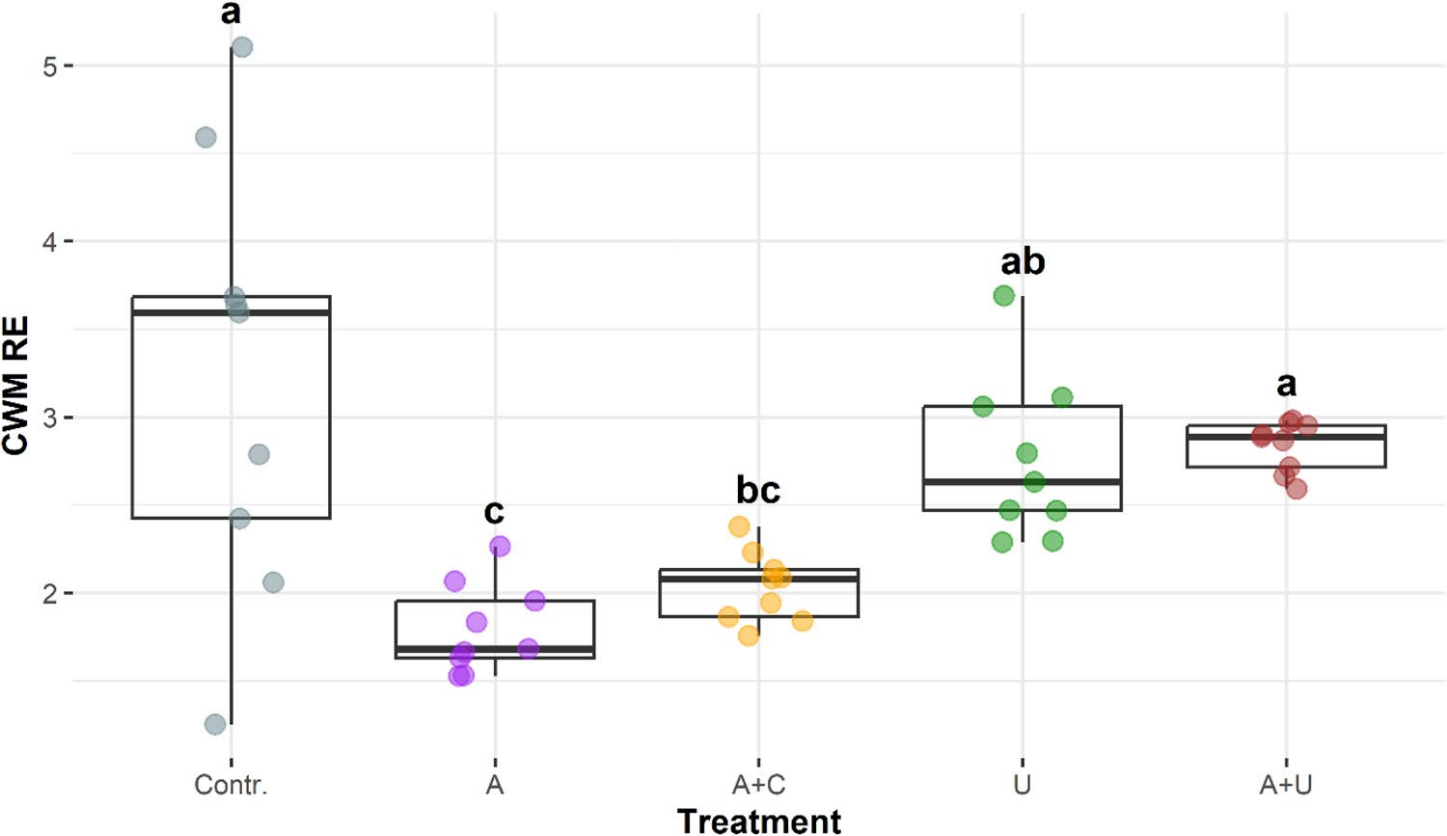
Community weighted means of relative elongation (CWM RE) values in the experimental setups from the 8th day of the experiment. The boxplots visually depict the distribution of data by representing the interquartile range with a box, indicating the median with a bold line inside the box, and displaying the minimum and maximum values through whiskers (points outside the whiskers’ range are outliers). Homogenous groups are indicated with different lowercase letters and based on significant differences of the CWM values of RE among treatments (Dunn’s test, P < 0.05). Abbreviations for experimental setups are specified under Fig. 3, (N = 45).

The size (GALD) and morphological traits (RE) exhibited similar distributional patterns among the five microcosm setups (Figs. 8, 9). The CWMs of GALD values showed the highest values and the greatest variability in the filtrated water (Contr.), while the lowest values occurred in the A microcosms that simulated the open water situation (Fig. 8). Similarly, low values characterised the microcosms with the artificial cotton-wool substrate (A+C). The means of CWMs were considerably higher in the microcosms with natural (*Utricularia*) substrates.

The pattern of CWM values of the relative elongation (RE) of the occurring species appeared to be similar to that of the GALD values detailed above (Fig. 9). However, due to the smaller dispersion of values the differences proved to be significant for the majority of paired comparisons.

We must note here that the species with large GALD and RE values were not identical in the F and A+U setups. Elongated euplanktic species (*Ankistrodesmus* sp.) occurred in the filtrated water (F), while in the U and A+U setups, where *Utricularia* plants were present benthic species constituted the majority of elongated elements of the assemblages.

**Figure 10:**
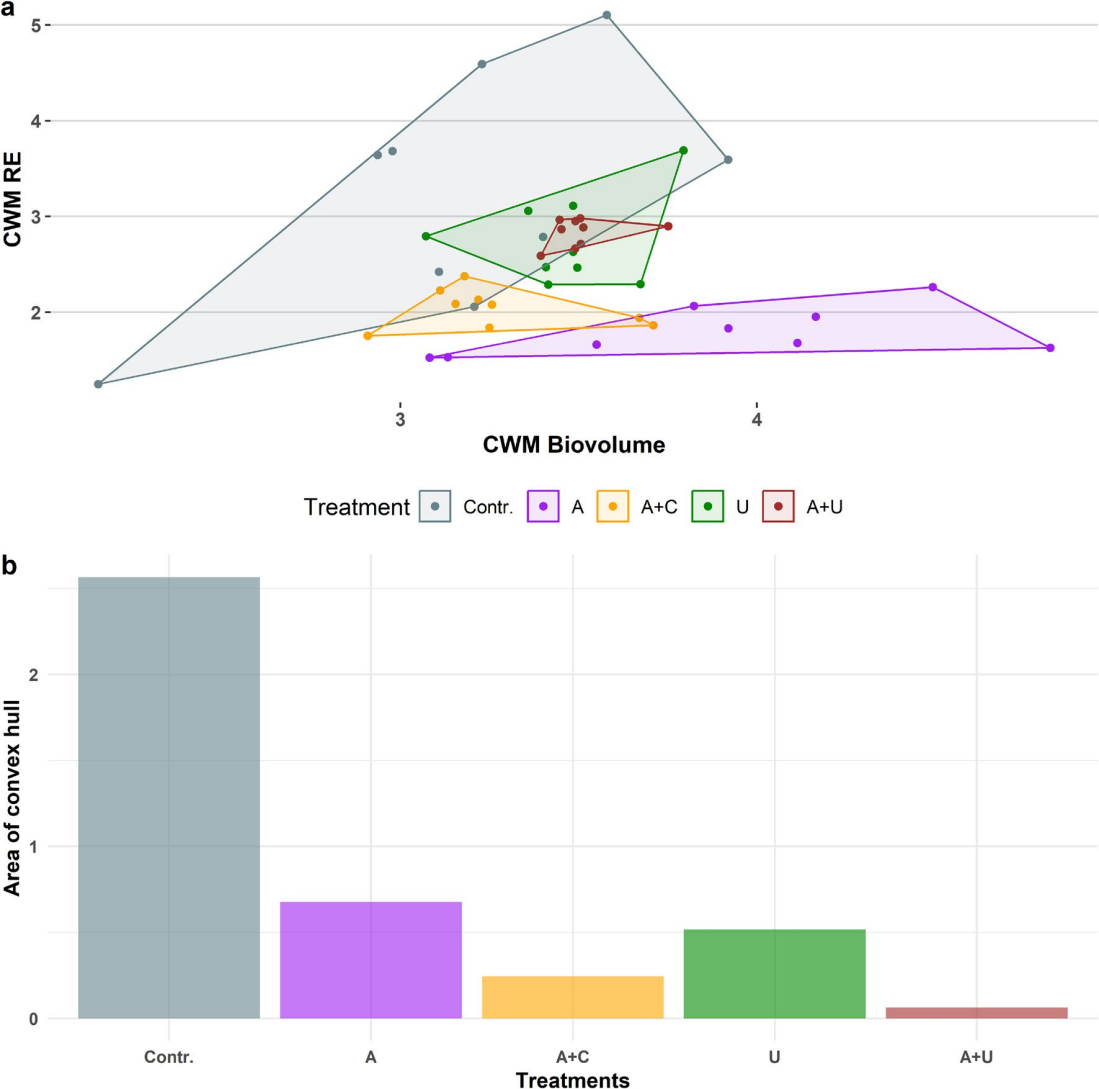
(**a**) Points of the samples of experimental setups in the relative elongation—biovolume morphospace from the 8th day of the experiment. Each treatments points are covered with polygons. (**b**) Areas of the polygons of the five experimental setups in the morphospace. Abbreviations for experimental setups are specified under Fig. 3, (N = 45).

The covered areas of the hulls (belonging to the five experimental setups—Fig. 10b) in the RE/Biovolume morphospace showed considerable differences (Fig. 10a). Large areas characterised the Contr. and A setups, that mimicked the open water habitats indicating large morphological and size variation of microalgae in the assemblages. The setups in which artificial and natural substrates were placed (A+C, U, A+U) had considerably smaller hull areas, referring to more stable assemblages in terms of the variation of their size and morphology.

## Discussion

In this study, we hypothesised that the complex three-dimensional artificial physical structures create specific microenvironments in the microcosms that is capable of sustaining unique microalgal assemblages, which differ from that of the open water and from that of the natural substrates (*Utricularia vulgaris*). To investigate this issue, we used microcosm experiments with cotton wool as artificial and *Utricularia vulgaris* as natural substrate. Both substrates performed well, because they maintained their physical structures during the entire study period, and decaying of their stems leaves or filaments did not happen throughout the study.

We observed 326 species in the algal inoculum of which 286 were non-diatoms. According to a recent review, 2489 species were detected in Hungary in the last 150 years^45^. Thus interestingly, a handful of submerged macrophytes and its immediate surroundings contain more than 10% of the regional microflora in a given moment, and as many species as can ordinarily be observed in a pond after many years of investigations^24,46^. This high species- and functional diversity of the inoculum enabled us to investigate how the various species and functions perform in different microcosm setups.

In line with our first hypothesis, we found significantly larger species richness in those microcosms in which artificial and natural substrates were placed compared to those that simulated the open water. However, this higher species richness did not coincide with higher functional richness values, which means that we have to reject our second hypothesis, which stated that the cotton-wool as an artificial solid physical structure would increase the habitat heterogeneity and thus, increases the number of functional niches in the water. However, these results are only seemingly controversial, because reduction in functional richness does not necessarily coincide with a decrease in the number of occurring species if functional redundancy of the FGs increases. This phenomenon was observed for F, J and N functional groups that showed considerably higher functional redundancy in the substrate containing microcosms. Reviewing hundreds of studies dealing with functional redundancy/ecosystem stability relationships Biggs et al.^47^ found positive relationship between the two system properties. The high functional redundancy of FGs in substrate containing systems implies that the extended macrovegetation in the littoral gives a stability to the microbial communities not only by providing habitats to functionally new assemblages^48^, but also by increasing functional redundancy of the existing FGs.

As it has been demonstrated in a pond enclosure experiment^25^, dynamics of the succession of metaphytic assemblages is similar to that observed in the case of the metaphyton. During the colonisation of solid substrates by benthic diatoms the most considerable changes take place within a few weeks, and in the subsequent days the more or less stabilized communities show much less alterations^49–51^. The observed changes in species and functional richness values showed similar patterns referring to similarities between developments of benthic and metaphytic assemblages. However, we must note here that the stabilized richness values do not mean that the assemblages remained constant, because species replacements occurred throughout the study period.

In accordance with our second hypothesis species and functional richness showed differences between the natural and artificial substrates. Although architectural complexity of the cotton wool provided as great morphological habitat complexity to the systems as did the *Utricularia*, species and functional richness of metaphyton in *Utricularia* containing microcosms was higher than those in which cotton wool was placed. This result was not trivial, because authors of a previous study^52^ found that filamentous algae had higher biovolume on artificial substrates than on submerged macrophytes. However, in our case, the results suggest that cotton wool has a more homogeneous structure than *Utricularia vulgaris*, with probably less habitat heterogeneity and fewer niches available. This explains why the cotton-wool assemblages had less functional richness than the *Utricularia* spp. containing assemblages.

There are more than sixty aquatic macrophytes species that are proven to be able to produce allelopathic substances that have negative effects on the adjacent microorganisms^53^. Thus, species- and functional richness of metaphyton in the *Utricularia* containing microcosms could even have been lower than in those containing cotton wool. However, the *Utricularia* spp. have two important characteristics: (i) they do not release allelopathic substances against microalgae, (ii) they have small traps to capture small prey animals. It seems that both features have positive effects on the development of metaphytic assemblages. Dos Santos et al.^27^ showed that *U*. *foliosa* provided favourable microenvironmental conditions for the development of periphytic assemblages. It has also been demonstrated by Płachno et al.^54^ that although several microorganisms (ciliates and algae) could be captured in the *Utricularia* traps, these creatures can survive in the hostile environment, and thus they can be considered as commensals rather than prey for these plants. During our sampling, we may have accidentally picked up individuals from the plants’ traps. This latter phenomenon explains why we observed higher richness values in those microcosms in which the *Utricularia* were placed in filtered lake water and did not contain the species rich inoculum.

Microalgae evolved a variety of morphological adaptations to cope with the constraints imposed by the physical properties of water^28^. In this experiment morphological differences among the occurring species have been expressed as the value of relative elongation. Elongation is an evolutionary response of microalgae to avoid sinking which is one of the primary reasons for the loss of species and biomass in lentic waters^55^. This was the reason why we hypothesised that morphological trait composition of the observed species would be different among the experimental setups. The filtrated lake water (used as control) showed by far the largest morphological variation in this study. Small sized spherical algae and elongated species with small and large GALD values dominated the samples of these microcosms. This result was not surprising, given that the “small” and the “elongated” are those traits that allow the microalgae to pass through the plankton net holes. The low morphological and size variations experienced in all microcosms that contained natural and artificial substrates however was inconsistent with our previous expectations. In accordance with previous findings^56,57^, we hypothesised that the larger habitat complexity coincides with larger morphological diversity of the resident species but we found contrasting results. The most plausible explanation is that the extremely low and high CWM values in the Contr., and A assemblages appeared because the small spherical and large elongated species occasionally dominated the assemblages, and although these species were present in the A+C, U, A+U microcosms, they showed lower abundance. Interestingly, there was no overlap in CWM based biomass vs. RE between the Contr. and A setups. The lack of overlap is mostly the result of different species composition (and therefore different morphological features in the community), because of the algal inoculum in the A setup. But it is also a result of the CWM method, because it gives back only one point per sample for the whole community, weighted by the relative abundance of algae with different morphologies and biomasses.

The values observed for the A+U and A+C samples were statistically farther away from the control than from each other, suggesting that the cotton wool mimicked the fine structure of submerged macrophytes to an extent.

The role of the littoral vegetation in maintaining microalgal diversity is understudied in the current literature, although similarly complex 3D habitat structures occur in other aquatic realms. Coral reefs are the most diverse aquatic ecosystems^58^ owing to their complex spatial structure that provides habitat for thousands of fish and invertebrate species. To restore the oceanic habitats damaged by climate change or anthropogenic actions creation of artificial corals has become a successful technology in the recent years^59^. Many studies demonstrated that the complex artificial 3D structures placed underwater helped to maintain the diversity of degraded oceanic habitats^60,61^. Although success of these restoration measures can be easily evaluated by assessing the species assemblages that can be detected by naked eye, microbial assemblages also invade and colonise these niches. Investigating the bacterial succession on artificial corals^62^ demonstrated that the colonization of bacteria on the artificial substrate is not a simple passive settlement from the water column, instead it is the result of a selection process driven by the physical and chemical structure of the surface. The observed high functional redundancy in the cotton wool containing microcosms demonstrate that the assemblages are not simple stochastic congregations of species, but rather a community of microalgae driven by selective forces of the complex physical structure.

Similarly to coral reefs that are one of the largest biogenic structures on Earth and possess a quarter of marine species^63,64^ aquatic macrophytes also create immense biogenic habitats in the lakes’ littoral, and host a great portion of microbial diversity of standing waters. Therefore, the knowledge that marine biologists accumulated on coral reef’s biota would be helpful in the understanding the role of littoral vegetation in controlling whole-lake’ microbial diversity.

## Conclusion

Submerged macrophytes create a complex three dimensional structure in the lakes’ littoral zone that serves an important habitat for both macro- and microscopic biota. The interaction of physical, chemical and biological features of this complex structure defines the habitat characteristics that select the species of resident assemblages. Given the role of microalgal assemblages as primary producers in aquatic food webs, their diversity and composition are vital for ecosystem stability and health. According to source-sink dynamics in metacommunity ecology, high diversity in metaphyton can enhance the diversity of associated phytoplankton. Furthermore, the diversity-stability relationship suggests that diverse microalgal assemblages, such as metaphyton and phytoplankton, contribute to the stability of higher trophic levels in the food web.

Understanding the organization of metaphytic assemblages in this unique habitat necessitates disentangling the various effects that drive their development and shape their composition and diversity. With the microcosm experiment we identified the similarities and differences in microalgal assemblages developed in aquaria simulating the environments of the open water, complex natural substrates and artificial physical substrates provided for the microalgae.

Differences in functional group and trait composition and diversity of the assemblages indicate that their development is not a stochastic process but rather driven by the specific niche characteristics of their habitats.

Our findings suggest that the assembly rules of metaphytic communities are critical for understanding the role of littoral aquatic macrovegetation in shaping the composition and diversity of microalgal assemblages in open water. This insight enhances our comprehension of ecosystem dynamics and can inform conservation and management strategies aimed at maintaining aquatic ecosystem health.

### Supplementary Information

Supplementary Figure 1.

Supplementary Figure 2.

Supplementary Figure 3.

Supplementary Tables.

Supplementary Legends.

## Data Availability

The datasets generated and/or analysed during the current study are available from the corresponding author on reasonable request.
