# Supplementary figures and images for: Physical structure of the environment contributes to the development of diversity of microalgal assemblages
Source: Sci Rep. 2024 Jun 12;14:13498. doi: 10.1038/s41598-024-63867-2 (PMC11169393; doi:10.1038/s41598-024-63867-2)

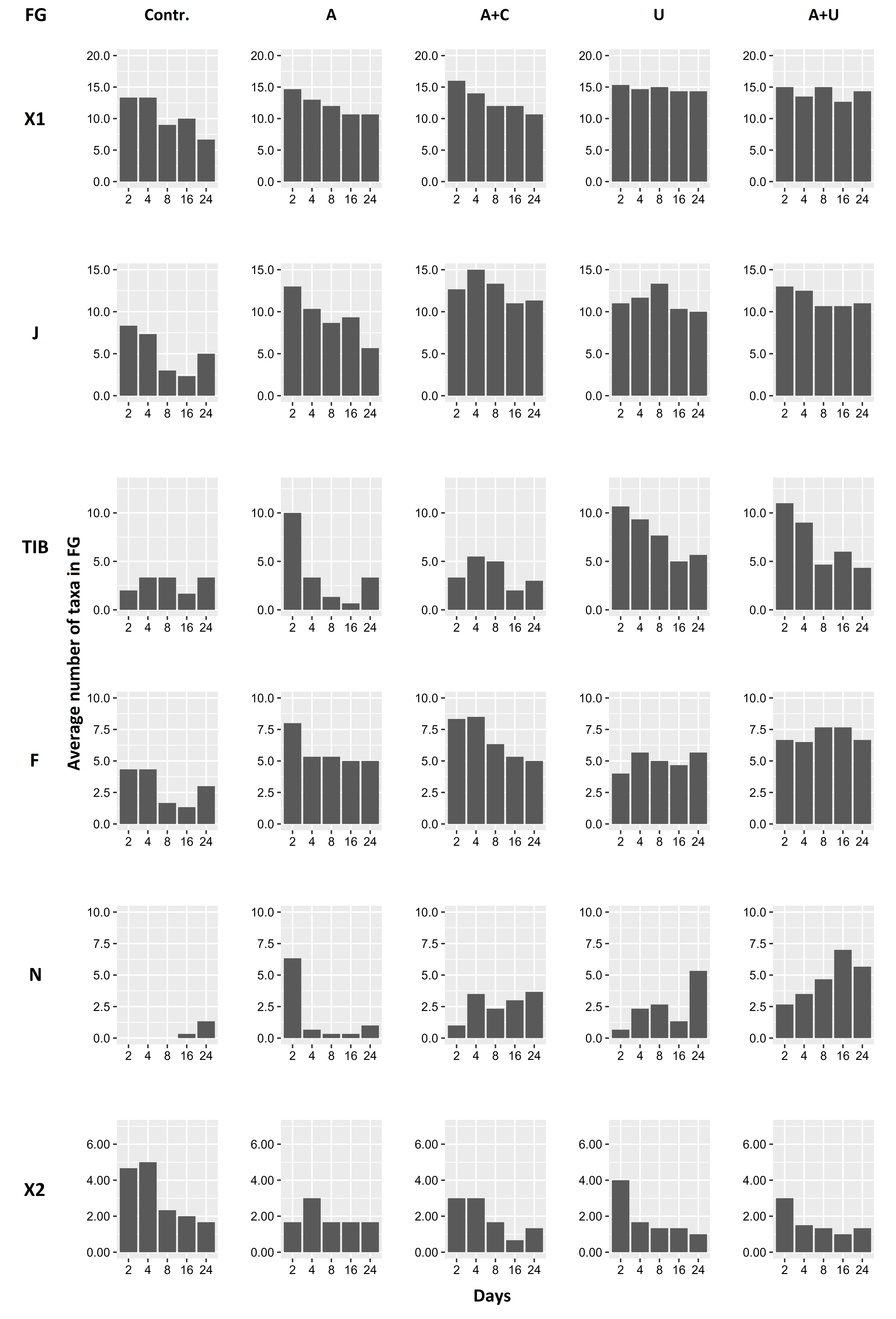

Supplement: Supplementary file 1 — Supplementary Figure 1. [file 41598_2024_63867_MOESM1_ESM.png]

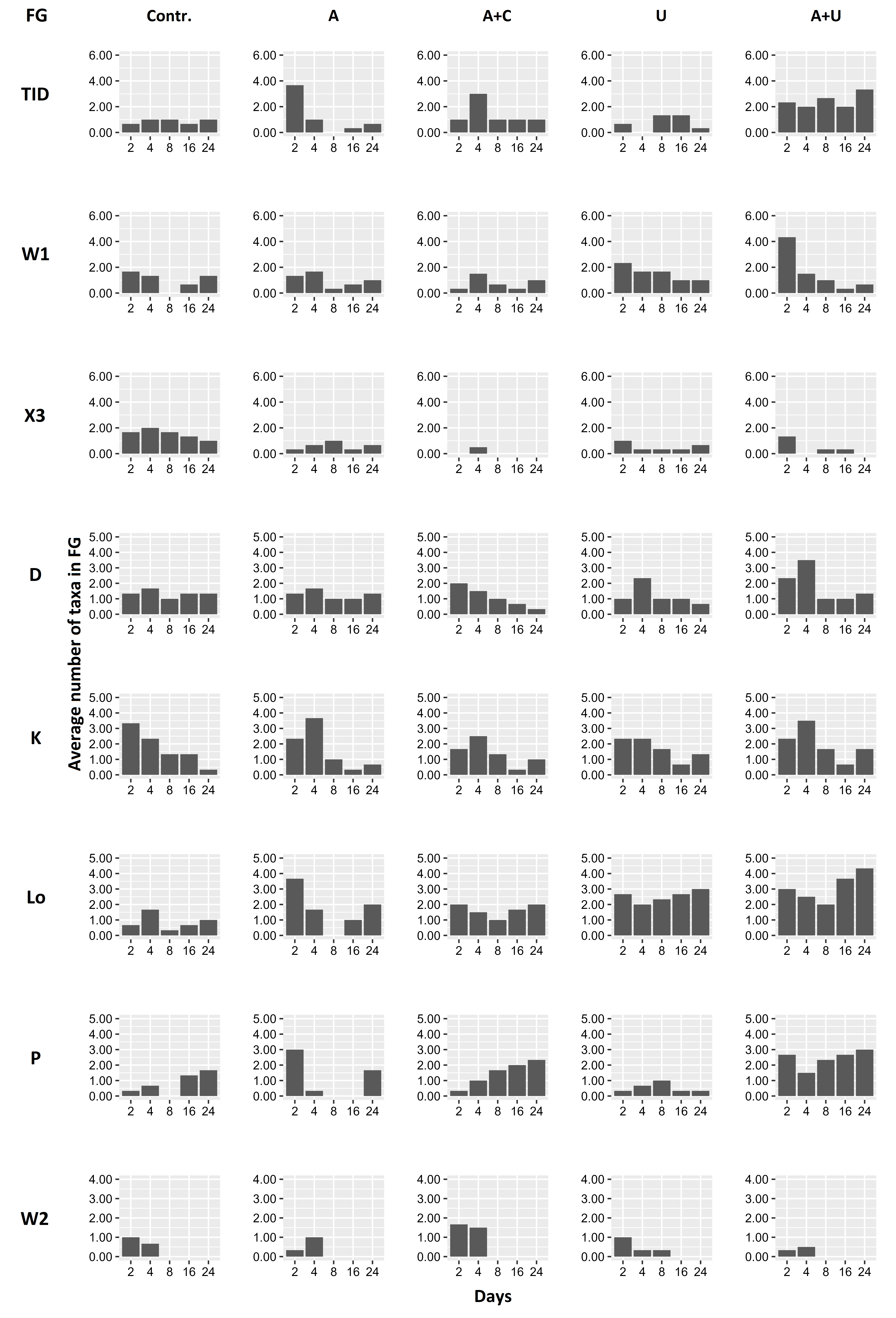

Supplement: Supplementary file 2 — Supplementary Figure 2. [file 41598_2024_63867_MOESM2_ESM.png]

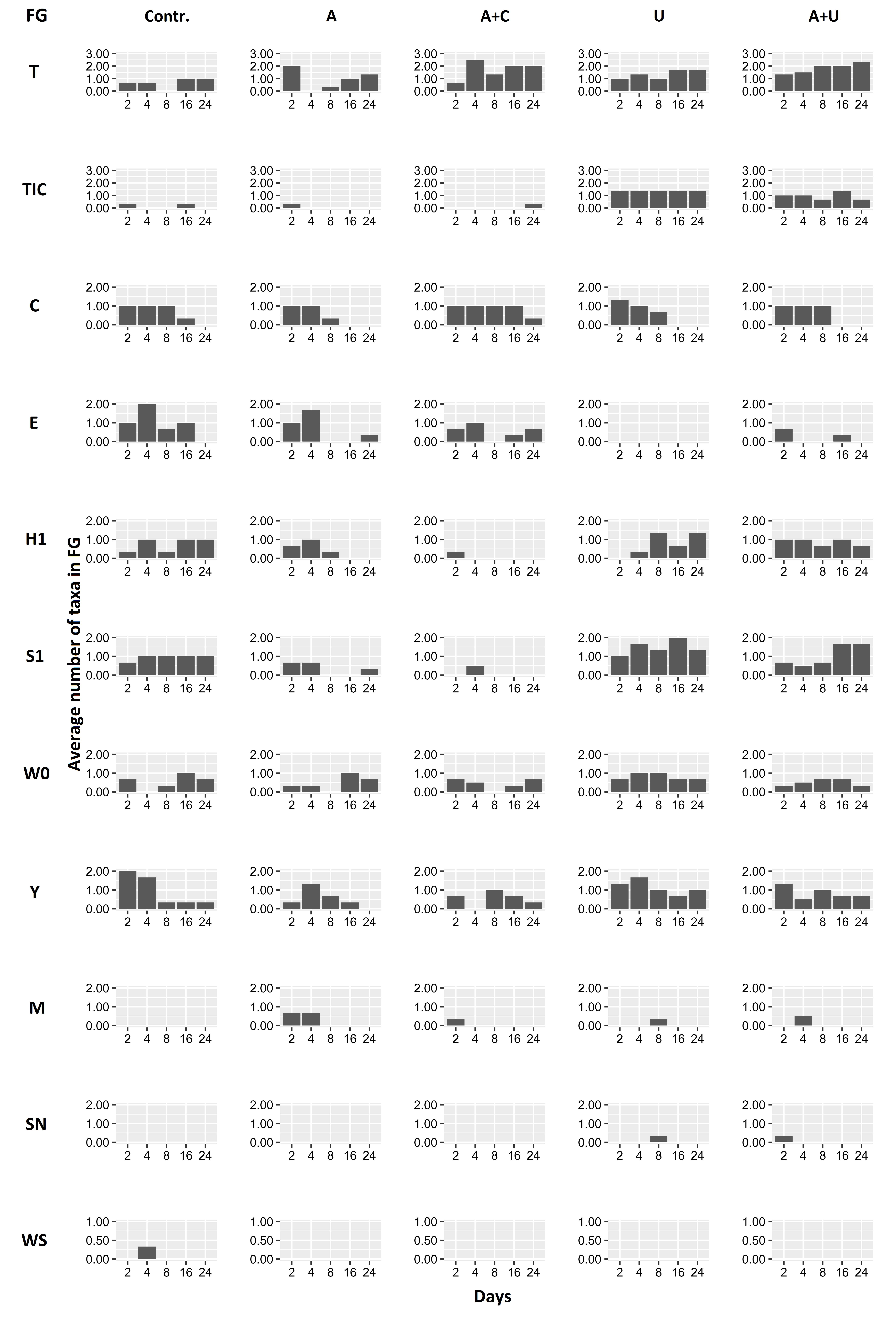

Supplement: Supplementary file 3 — Supplementary Figure 3. [file 41598_2024_63867_MOESM3_ESM.png]
