# Supplementary material for: Physical structure of the environment contributes to the development of diversity of microalgal assemblages
Source: Sci Rep. 2024 Jun 12;14:13498. doi: 10.1038/s41598-024-63867-2 (PMC11169393; doi:10.1038/s41598-024-63867-2)
Supplement: Supplementary file 4 — Supplementary Tables. [file 41598_2024_63867_MOESM4_ESM.docx]

Supplementary Table 1: Physical and chemical properties of the oxbow lake at sampling time, when materials were collected.

| Physical and chemical properties at the time of sampling (29.07.2019) | |
| --- | --- |
| Water temperature (^○^C) | 26.4 |
| Secchi transparency (m) | 0.5 |
| pH | 8.09 |
| Electrical Conductivity (µScm^-1^) | 484 |
| Dissolved oxygen (mg L^-1^) | 5.45 |
| Oxygen saturation (%) | 67.3 |
| Chlorophyll-a (mg L^-1^) | 71 |
| Ammonium (µg L^-1^) | 330 |
| Nitrate (µg L^-1^) | 400 |
| Nitrite (µg L^-1^) | 5.0 |
| Total Nitrogen (µg L^-1^) | 1,700 |
| Ortophosphate (µg L^-1^) | 80 |
| Total phosphorus (µg L^-1^) | 170 |
| Silica (µg L^-1^) | 664 |

Supplementary Table 2: Results of linear models of different time intervals vs number of taxa

| **Time interval in days** | **p-value of time in model** | **Significance** |
| --- | --- | --- |
| 0-24 | 2.64E-05 | significant |
| 0-8 | 1.21E-07 | significant |
| 8-24 | 0.441687 | non-significant |

Supplementary Table 3: The observed taxa, their functional groups (FG) and their presence in the different experimental setups. Abbreviations for experimental setups are specified under Fig. 3.

|  | **FG** | **Contr.** | **A** | **A+C** | **U** | **A+U** |
| --- | --- | --- | --- | --- | --- | --- |
| *Achnanthes* sp. | TIB |  |  |  |  | X |
| *Achnanthidium minutissimum* | TIB | X |  |  | X | X |
| *Acutodesmus acutiformis* | J | X |  | X | X | X |
| *Amphikrikos minutissimus* | K | X | X | X |  | X |
| *Amphipleura pellucida* | TIB | X | X |  |  | X |
| *Amphora pediculus* | TIB | X |  |  |  |  |
| *Anabaena* sp. | H1 | X | X | X | X | X |
| *Ankistrodesmus densus* | X1 | X | X | X | X | X |
| *Ankistrodesmus falcatus* | X1 | X |  |  |  |  |
| *Aphanizomenon issatchenkoi* | H1 | X |  |  | X |  |
| *Aphanizomenon* sp. | H1 | X | X | X | X | X |
| *Aphanocapsa delicatissima* | K | X | X | X | X | X |
| *Aphanocapsa holsatica* | K | X | X | X | X | X |
| *Aphanocapsa parasitica* | K | X |  |  |  |  |
| *Aphanothece nidulans* | K | X |  | X |  |  |
| *Aphanothece* sp. | K | X | X | X | X | X |
| *Aulacoseira distans* | C | X |  |  | X |  |
| *Aulacoseira granulata* | P |  | X |  |  |  |
| *Bicosoeca planctonica* | X3 |  | X | X |  | X |
| *Binuclearia lauterbornii* | T | X |  | X | X |  |
| *Botryococcus braunii* | F |  | X | X | X | X |
| *Calothrix braunii* | TIC |  | X | X | X | X |
| *Carteria radiosa* | W0 |  |  |  | X |  |
| *Centrales* (15 μm) | D |  |  |  |  | X |
| *Centrales* (20 μm) | D |  |  |  |  | X |
| *Centrales* (5 μm) | D | X | X | X | X | X |
| *Characium ornithocephalum* | X1 |  | X |  | X |  |
| *Characium* sp. | X1 | X | X |  | X | X |
| *Chlamydomonas reinhardtii* | W0 |  |  | X |  | X |
| *Chlamydomonas* sp. (small cell) | W0 |  |  |  | X |  |
| *Chlamydomonas* sp. | W0 | X | X | X | X | X |
| *Chlorella* sp. | X1 | X | X | X | X | X |
| *Chlorotetraedron* *incus* | X1 | X | X | X | X | X |
| *Chromulina* sp. | X2 | X | X | X | X | X |
| *Chroococcus aphanocapsoides* | Lo |  |  |  |  | X |
| *Chroococcus limneticus* | Lo | X | X | X | X | X |
| *Chroococcus* sp. | Lo |  |  |  |  | X |
| *Chroococcus turgidus* | Lo | X | X | X | X | X |
| *Chrysococcus rufescens* | X3 | X | X | X | X | X |
| *Closterium acerosum* | TID |  | X |  |  |  |
| *Closterium aciculare* | P |  |  |  | X |  |
| *Closterium acutum* | P | X | X |  | X |  |
| *Closterium acutum* var. *variabile* | P | X | X | X |  | X |
| *Closterium dianae* | TID |  |  |  | X | X |
| *Closterium idiosporum* | TID |  |  |  |  | X |
| *Closterium incurvum* | TID | X | X | X | X | X |
| *Closterium pronum* | TID | X | X | X | X | X |
| *Closterium* sp. | TID | X |  |  |  | X |
| *Closterium tumidulum* | TID |  | X | X |  | X |
| *Cocconeis placentula* | TIB |  | X |  | X | X |
| *Coelastrum indicum* | J |  |  |  |  | X |
| *Coelastrum pseudomicroporum* | J | X | X | X | X | X |
| *Coelastrum reticulatum* | J |  | X | X |  | X |
| *Coelomoron pusillum* | K | X | X | X | X | X |
| *Coelosphaerium* sp. | Lo |  |  |  |  | X |
| *Coenochloris helvetica* | J |  |  |  | X |  |
| *Cosmarium botrytis* | N |  | X |  | X | X |
| *Cosmarium contractum* | N |  |  | X |  | X |
| *Cosmarium contractum* var. *rotundatum* | N |  | X |  |  | X |
| *Cosmarium crenulatum* | N | X | X |  | X | X |
| *Cosmarium dickii* | N |  |  |  |  | X |
| *Cosmarium fastidiosum* | N |  | X |  |  |  |
| *Cosmarium humile* | N |  | X |  |  |  |
| *Cosmarium kjellmanii* | N | X |  |  |  |  |
| *Cosmarium moniliforme* | N |  |  |  | X |  |
| *Cosmarium notatum* | N | X |  |  |  |  |
| *Cosmarium phaseolus* | N | X | X | X | X | X |
| *Cosmarium polygonatum* | N |  | X | X | X | X |
| *Cosmarium pseudoretusum* var. *inaequalipellicum* | N |  |  |  |  | X |
| *Cosmarium pseudoretusum* var. *pseudoretusum* | N |  | X |  |  |  |
| *Cosmarium pseudowambaerense* | N |  |  |  | X |  |
| *Cosmarium punctulatum* | N |  |  | X |  | X |
| *Cosmarium pygmaeum* | N |  | X | X |  | X |
| *Cosmarium regnelii* var. *regnelii* | N | X | X | X | X | X |
| *Cosmarium regnesii* | N |  |  |  | X | X |
| *Cosmarium regnesii* var. *regnesii* | N | X |  |  |  |  |
| *Cosmarium reniforme* | N |  | X |  |  | X |
| *Cosmarium sexnotatum* | N |  | X | X | X | X |
| *Cosmarium* sp. (36 μm) | N |  |  | X |  |  |
| *Cosmarium* sp. | N |  |  |  | X |  |
| *Cosmarium subcostatum* | N |  |  |  | X |  |
| *Cosmarium subgranatum* | N |  |  |  | X | X |
| *Cosmarium subprotumidum* | N |  | X |  | X | X |
| *Cosmarium subprotumidum* var. *pyramidale* | N |  |  |  | X | X |
| *Cosmarium subtumidum* | N |  |  |  |  | X |
| *Cosmarium tenue* | N | X | X | X | X | X |
| *Cosmarium undulatum* | N |  |  |  | X |  |
| *Cosmocladium saxonicum* | N |  | X |  |  |  |
| *Crucigenia tetrapedia* | X1 | X | X | X | X | X |
| *Cryptomonas marssonii* | Y | X | X | X | X | X |
| *Cryptomonas obovata* | Y | X | X | X | X | X |
| *Cryptomonas ovata* | Y | X |  |  |  |  |
| *Cryptomonas* sp. | Y |  |  |  |  | X |
| *Cyanocatenula calyptrata* | K |  | X |  |  |  |
| *Cyanogranis ferruginea* | K | X | X | X | X | X |
| *Cylindrospermopsis raciborskii* | SN |  |  |  | X |  |
| *Cymbella* sp. | TIB | X | X | X | X | X |
| *Dactylosphaerium jurisii* | J | X | X | X | X | X |
| *Desmatractum bipyramidatum* | J |  | X | X |  | X |
| *Desmatractum indutum* | J |  |  |  | X | X |
| *Desmodesmus subspicatus* | J | X |  |  | X | X |
| *Diatoma* sp. | TIB |  |  |  |  | X |
| *Dictyosphaerium ehrenbergianum* | F | X | X | X | X | X |
| *Dictyosphaerium pulchellum* | F |  |  | X |  | X |
| *Dimorphococcus lunatus* | F | X | X | X |  | X |
| *Dinobryon bavaricum* | E | X | X | X | X | X |
| *Dinobryon sertularia* | E |  | X | X |  |  |
| *Dinobryon* sp. | E |  |  | X |  |  |
| *Diplochloris lunata* | J |  | X |  |  |  |
| *Diploneis* sp. | TIB | X |  |  |  |  |
| *Dolichospermum smithii* | H1 |  |  |  |  | X |
| *Elakatothrix genevensis* | F | X |  |  |  |  |
| *Elakatothrix* sp. | F | X | X | X | X | X |
| *Elakatothrix spirochroma* | F | X |  |  |  |  |
| *Epipyxis* sp. | E |  | X | X | X | X |
| *Epithemia adnata* | TIB | X | X | X | X | X |
| *Epithemia gibba* | TIB | X |  |  |  |  |
| *Epithemia sorex* | TIB |  |  |  | X | X |
| *Epithemia* sp. | TIB |  | X | X | X | X |
| *Euastrum denticulatum* | TID |  |  |  |  | X |
| *Euastrum germanicum* | TID |  |  |  | X | X |
| *Euglena acus* | W1 |  | X | X |  | X |
| *Euglena clavata* | W1 |  |  |  |  | X |
| *Euglena ehrenbergii* | W1 |  |  |  |  | X |
| *Euglena gracilis* | W1 |  |  |  | X | X |
| *Euglena pisciformis* | W1 | X | X | X | X | X |
| *Euglena polymorpha* | W1 | X |  |  | X | X |
| *Euglena* sp. | W1 |  | X | X |  | X |
| *Euglena texta* | W1 | X |  |  | X | X |
| *Euglena variabilis* | W1 |  |  |  | X | X |
| *Euglena viridis* | W1 | X | X | X | X | X |
| *Eutetramorus planctonicus* | J | X | X |  | X | X |
| *Fragilaria capucina* | TIB |  | X | X |  |  |
| *Fragilaria crotonensis* | TIB | X | X | X | X | X |
| *Fragilaria* sp. | TIB | X | X | X |  |  |
| *Fragilaria tenera* var. *nanana* | TIB |  | X | X | X | X |
| *Golenkinia radiata* | X1 |  | X |  |  |  |
| *Gomphonema acuminatum* | TIB | X |  |  | X | X |
| *Gomphonema augur* | TIB |  |  |  | X |  |
| *Gomphonema gracile* | TIB | X | X | X | X | X |
| *Gomphonema parvulum* | TIB |  | X | X | X | X |
| *Gomphonema* sp. | TIB |  | X |  | X | X |
| *Gomphonema truncatum* | TIB |  |  |  |  | X |
| *Gomphosphaeria* sp. | Lo | X |  |  |  | X |
| *Gonatozygon brebissonii* | P |  |  |  | X | X |
| *Gonatozygon monotaenium* | P | X | X | X | X | X |
| *Goniochloris mutica* | J |  |  |  |  | X |
| *Granulocystopsis helenae* | F | X | X |  |  |  |
| *Gymnodinium* sp. | Lo | X |  |  |  |  |
| *Hippodonta capitata* | TIB |  |  |  |  | X |
| *Jaaginema* sp. | S1 |  | X | X |  |  |
| *Kephyrion cordatum* | X2 |  | X | X |  | X |
| *Kephyrion litorale* | X2 |  | X | X |  | X |
| *Kephyrion rubri-claustri* | X2 |  | X |  | X |  |
| *Kephyrion* sp. | X2 |  |  |  | X |  |
| *Kephyrion spirale* | X2 |  |  | X |  |  |
| *Kirchneriella aperta* | F | X | X | X | X | X |
| *Kirchneriella contorta* | F | X | X | X | X | X |
| *Kirchneriella irreguralis* | F | X | X | X | X | X |
| *Kirchneriella obesa* | F |  |  |  | X |  |
| *Klebsormidium* sp. | T | X |  | X | X |  |
| *Koliella longiseta* | X3 | X | X | X | X | X |
| *Koliella planctonica* | X3 | X | X | X | X | X |
| *Koliella tenuis* | X3 | X | X | X | X | X |
| *Lagerheimia ciliata* | X1 | X | X |  | X |  |
| *Lagerheimia minor* | X1 | X |  |  |  | X |
| *Lagerheimia subsalsa* | X1 | X | X | X | X | X |
| *Lepocinclis ovum* | W1 |  |  |  | X |  |
| *Lobomonas ampla* | W1 |  |  |  | X |  |
| *Lyngbya martensiana* | TIC | X | X | X | X | X |
| *Lyngbya* sp. | TIC | X | X | X | X | X |
| *Merismopedia glauca* | Lo |  | X |  |  |  |
| *Messastrum gracile* | X1 |  |  |  | X |  |
| *Micractinium belenophorum* | J | X | X |  | X |  |
| *Micractinium pusillum* | X1 |  |  | X |  | X |
| *Microcystis smithii* | M |  | X |  | X |  |
| *Microcystis* sp. | M |  |  |  | X |  |
| *Monas arhabdomonas* | X3 | X | X | X | X | X |
| *Monoraphidium arcuatum* | X1 |  | X |  |  | X |
| *Monoraphidium circinale* | X1 | X | X | X | X | X |
| *Monoraphidium contortum* | X1 |  | X | X |  | X |
| *Monoraphidium convolutum* | X1 | X | X | X | X | X |
| *Monoraphidium griffithii* | X1 |  |  |  | X |  |
| *Monoraphidium komarkovae* | X1 | X | X | X | X | X |
| *Monoraphidium minutum* | X1 | X | X | X | X | X |
| *Monoraphidium tortile* | X1 |  |  |  | X |  |
| *Mougeotia* div. sp. | T | X | X | X | X | X |
| *Mougeotia* sp. | T |  |  |  |  | X |
| *Navicula capitatoradiata* | TIB | X | X | X | X | X |
| *Navicula cryptocephala* | TIB |  |  |  | X | X |
| *Navicula cryptotenella* | TIB | X |  |  |  |  |
| *Navicula cuspidata* | TIB | X |  |  | X |  |
| *Navicula gracilis* | TIB |  |  |  | X |  |
| *Navicula lanceolata* | TIB | X | X | X | X | X |
| *Navicula radiosa* | TIB | X |  |  | X |  |
| *Navicula* sp. | TIB | X | X | X | X | X |
| *Navicula veneta* | TIB | X | X | X | X | X |
| *Neglectella solitaria* | F | X | X | X |  |  |
| *Nephrochlamys allanthoidea* | X2 | X | X | X | X | X |
| *Nephrochlamys subsolitaria* | X2 | X | X | X | X | X |
| *Nephrocytium limneticum* | X2 |  |  |  | X |  |
| *Nitzschia dissipata* | TIB | X | X | X | X | X |
| *Nitzschia gracilis* | TIB | X | X | X | X | X |
| *Nitzschia intermedia* | TIB | X | X | X |  |  |
| *Nitzschia linearis* | TIB | X | X | X | X | X |
| *Nitzschia palea* | TIB | X | X | X | X | X |
| *Nitzschia reversa* | D |  |  | X |  | X |
| *Nitzschia* sp. | TIB | X | X | X | X | X |
| *Onychonema filiforme* | TID | X | X | X | X | X |
| *Oocystis marssonii* | F | X |  |  |  |  |
| *Oocystis* sp. | F |  | X |  |  | X |
| *Oscillatoria annae* | S1 |  |  |  | X | X |
| *Oscillatoria limnetica* | S1 | X | X | X | X | X |
| *Oscillatoria* sp. | TIC | X | X | X | X | X |
| *Pachycladella komarekii* | X1 | X | X | X |  | X |
| *Palmelloid sphere* | W1 |  | X | X |  | X |
| *Pandorina smithii* | G |  | X |  |  | X |
| *Pediastrum boryanum* | J | X | X | X |  | X |
| *Pediastrum duplex* | J |  |  |  |  | X |
| *Pediastrum duplex* var. *gracilimum* | J |  | X | X |  |  |
| *Pediastrum simplex* | J | X | X | X | X | X |
| *Pediastrum tetras* | J |  | X |  |  |  |
| *Penium margaritaceum* | TID |  | X | X |  | X |
| *Peridiniopsis* sp. | Lo |  |  | X |  |  |
| *Peridinium bipes* | Lo | X | X | X | X | X |
| *Peridinium* sp. | Lo | X | X |  |  | X |
| *Phacus acuminatus* | W1 |  | X | X |  | X |
| *Phacus agilis* | W1 | X |  |  | X | X |
| *Phacus glaber* | W1 |  |  |  | X |  |
| *Phacus granum* | W1 | X |  |  |  |  |
| *Phacus lismorensis* | W1 |  |  |  | X |  |
| *Phacus longicauda* | W1 |  | X | X |  | X |
| *Phacus longicauda* var. *tortuosus* | W1 |  | X |  |  | X |
| *Phacus orbicularis* | W1 |  |  |  |  | X |
| *Phacus parvulus* | W1 |  |  |  | X |  |
| *Phacus polytrophos* | W1 | X |  |  |  |  |
| *Phacus raciborskii* | W1 | X | X | X | X | X |
| *Phacus wettsteinii* | W1 |  |  |  | X |  |
| *Phormidium* sp. | TIC |  |  |  | X |  |
| *Pinnularia microstauron* | TIB |  |  |  | X |  |
| *Pinnularia* sp. | TIB | X | X | X |  | X |
| *Plagioselmis lacustris* | X2 | X | X | X | X | X |
| *Plagioselmis nannoplanktonica* | X2 |  | X | X | X | X |
| *Planktolyngbya limnetica* | S1 |  |  |  | X |  |
| *Planktolyngbya* spp. | S1 | X |  |  | X |  |
| *Planothidium lanceolatum* | TIB | X | X | X | X | X |
| *Pseudanabaena* sp. | S1 | X | X | X | X | X |
| *Pseudodidymocystis inconspicua* | X1 | X | X | X |  | X |
| *Pseudokephyrion entzii* | X3 |  | X | X |  | X |
| *Pseudokephyrion minutissimum* | X3 |  |  |  | X |  |
| *Pseudopediastrum subgranulatum* | J |  |  | X | X | X |
| *Pseudostaurastrum limneticum* | X1 | X | X | X | X | X |
| *Pseudotetraedriella kamillae* | X1 |  | X | X | X | X |
| *Radiophilum mesomorphum* | T |  |  |  |  | X |
| *Raphidiopsis mediterranea* | SN |  |  |  |  | X |
| *Rhoicosphaenia abbreviata* | TIB | X | X | X | X | X |
| *Scenedesmus acuminatus* | J | X | X | X | X | X |
| *Scenedesmus acutiformis* | J | X | X | X | X | X |
| *Scenedesmus acutus* | J | X | X | X | X | X |
| *Scenedesmus arcuatus* | J |  | X |  | X | X |
| *Scenedesmus armatus* | J |  |  |  |  | X |
| *Scenedesmus communis* | J | X | X | X | X | X |
| *Scenedesmus ecornis* | J | X | X | X | X | X |
| *Scenedesmus grahneisii* | J | X | X | X | X | X |
| *Scenedesmus intermedius* | J | X | X | X | X | X |
| *Scenedesmus juvenilis* | J | X | X | X | X | X |
| *Scenedesmus obtusus* | J | X |  |  |  |  |
| *Scenedesmus opoliensis* | J | X | X | X | X | X |
| *Scenedesmus ovalternus* | J | X | X | X | X | X |
| *Scenedesmus protuberans* | J |  |  |  |  | X |
| *Scenedesmus* sp. | J |  |  | X |  |  |
| *Scenedesmus spicatus* | J | X |  |  |  |  |
| *Scenedesmus spinosus* | J | X | X | X | X | X |
| *Schroederia setigera* | J | X | X | X | X | X |
| *Selenastrum bibraianus* | X1 |  | X | X | X | X |
| *Siderocelis ornata* | X1 | X |  |  |  | X |
| *Skeletonema potamos* | D | X | X | X |  | X |
| *Snowella lacustris* | Lo | X |  |  |  | X |
| *Snowella litoralis* | Lo |  | X |  | X | X |
| *Snowella* sp. | Lo |  | X | X | X | X |
| *Sorastrum spinulosum* | J | X |  |  |  |  |
| *Sphaerellopsis aulata* | X3 |  |  |  | X |  |
| *Sphaerozosma vertebratum* | TID |  |  |  | X |  |
| *Spirogyra* sp. | TID | X | X | X | X | X |
| *Spondylosium planum* | N | X | X | X | X | X |
| *Staurastrum boreale* | P |  | X |  |  | X |
| *Staurastrum* *manfeldtii* var. *manfeldtii* | P | X |  | X |  | X |
| *Staurastrum micron* | P |  |  |  |  | X |
| *Staurastrum oxyacanthum* | N | X |  |  | X | X |
| *Staurastrum paradoxum* | P |  |  |  | X | X |
| *Staurastrum polymorphum* | P |  | X | X |  | X |
| *Staurastrum punctulatum* | P | X | X | X | X | X |
| *Staurastrum* sp. | N | X | X | X | X | X |
| *Staurastrum tetracerum* | P |  | X | X | X | X |
| *Staurodesmus* *cuspidatus* var. *cuspidatus* | N | X | X |  |  | X |
| *Staurodesmus dejectus* | P | X | X | X | X | X |
| *Staurodesmus dickiei* | P | X | X | X | X | X |
| *Stephanocyclus menenghinianus* | C | X |  | X | X |  |
| *Stichococcus contortus* | X2 | X | X | X | X | X |
| *Stichococcus pelagicus* | X2 | X | X |  | X | X |
| *Stichococcus sp.* | X2 | X | X | X |  | X |
| *Synura* sp. | WS |  | X | X |  | X |
| *Teilingia granulata* | TID |  | X | X | X |  |
| *Tetradesmus obliquus* | J |  |  | X |  | X |
| *Tetraedriella regularis* | X1 |  | X | X |  | X |
| *Tetraedriella* sp. | X1 |  |  |  | X |  |
| *Tetraedriella spinigera* | X1 | X | X | X | X | X |
| *Tetraedron caudatum* | X1 | X | X | X | X | X |
| *Tetraedron minimum* | X1 | X | X | X | X | X |
| *Tetraedron triangulare* | X1 | X |  |  | X |  |
| *Tetrastrum heteracanthum* | X1 | X | X | X | X | X |
| *Tetrastrum triangulare* | X1 |  |  | X |  |  |
| *Trachelomonas hispida* | W2 |  | X | X |  | X |
| *Trachelomonas intermedia* | W2 |  | X | X | X | X |
| *Trachelomonas oblonga* | W2 | X | X | X | X | X |
| *Trachelomonas planctonica* | W2 | X | X | X | X | X |
| *Trachelomonas volvocinopsis* | W2 | X | X | X |  | X |
| *Trachydiscus lenticularis* | X2 | X | X | X | X | X |
| *Treubaria triappendiculata* | X1 | X | X | X | X | X |
| *Ulnaria acus* | D | X | X | X | X |  |
| *Ulnaria capitata* | TIB |  | X | X |  | X |
| *Ulnaria ulna* | TIB | X | X | X | X | X |
| *Ulothrix* sp. | T | X | X | X | X | X |
| *Willea crucifera* | X1 |  | X | X |  | X |
| *Woronichinia naegeliana* | Lo |  |  |  |  | X |
| *Xanthidium variabile* | N |  |  |  |  | X |
