# Supplementary material for: Physical structure of the environment contributes to the development of diversity of microalgal assemblages
Source: Sci Rep. 2024 Jun 12;14:13498. doi: 10.1038/s41598-024-63867-2 (PMC11169393; doi:10.1038/s41598-024-63867-2)
Supplement: Supplementary file 5 — Supplementary Legends. [file 41598_2024_63867_MOESM5_ESM.docx]

Supplementary Fig. 1: Changes of functional redundancy values (as average number of functional groups - FGs) of the experimental setups through the study period. Abbreviations for setups are specified under Fig. 3.
